# A phase 1/PK study of Sunitinib with highly active antiretroviral therapy (HAART) in HIV+ patients with solid tumors: AIDS malignancy consortium study 061

**DOI:** 10.1186/1750-9378-7-S1-O15

**Published:** 2012-04-19

**Authors:** John F Deeken, Michelle A Rudek, Page C Moore, David Aboulafia, Ryan Sullivan, John Gerecitano, Mary Cianfrocca, David Henry, Lee Ratner, Bruce Dezube, Kimberly Mosby, Melinda Tibbals, Richard F Little, S P Ivy, Ronald T Mitsuyasu

**Affiliations:** 1 Lombardi Comprehensive Cancer Center, Georgetown University, Washington, DC, USA; 2 The Sidney Kimmel Comprehensive Cancer Center at Johns Hopkins University, Baltimore, MD, USA; 3 University of Arkansas for Medical Sciences, Little Rock, AR, USA; 4 Virginia Mason Medical Center, Seattle, WA, USA; 5 Beth Israel Deaconess Medical Center, Boston, MA, USA; 6 Memorial Sloan-Kettering Cancer Center, New York, NY, USA; 7 Northwestern University, Chicago, IL, USA; 8 Pennsylvania Hospital, Philadelphia, PA, USA; 9 Washington University, St. Louis, MO, USA; 10 EMMES Corporation, Rockville, MD, USA; 11 National Cancer Institute, National Institutes of Health, Rockville, MD, USA; 12 University of California, Los Angeles Care Center, Los Angeles, CA, USA

## Background

In developed counties the rates of non-AIDS defining cancers (NADCs) now exceed those of AIDS-defining cancers in HIV-positive patients. Drug-drug interactions between HAART and chemotherapy may complicate the treatment of patients with NADCs. In order to determine the proper dosing of new targeted chemotherapies in patients with NADCs who are also on HAART, the AMC is performing a series of phase I/pharmacokinetic (PK) studies to determine the proper dosing of these agents in HIV+ cancer patients. We present the results of the first such study which investigated sunitinib, an oral multiple tyrosine kinase inhibitor.

## Methods

Patients with HIV and cancers refractory to standard therapy were stratified into two arms: (1) non-ritonavir based HAART or (2) ritonavir-based HAART. Six patients were to be enrolled on arm 1 and receive the standard dose of sunitinib (50mg po qd). Arm 2 used a phase I, 3+3 dose escalation design (25, 37.5, and 50mg po qd). Cycles were 4 weeks on/2 weeks off. PK monitoring of sunitinb and its active metabolite (S-M) were performed throughout cycle one, and normalized based on dose level, to calculate AUC_0-24_, C_max_, and trough level.

## Results

Between 8/09 and 4/11, 19 patients were enrolled and completed cycle 1 (10 on arm 1, 9 on arm 2). Cancer types included Kaposi’s Sarcoma (7), lung (2), anal (2), head and neck (2), NHL (1), and other solid tumors (5). Median cycles was 2 (range 1-7). Following enrollment of the first 6 patients to Arm 1, that arm was expanded to include 3 additional patients on efavirenz, a potent inducer of CYP3A4, to better characterize sunitinb-efavirenz interactions. Patients on arm 1 tolerated standard treatment of 50mg with no dose limiting toxicities (DLTs). In the ritonavir arm, three patients tolerated 25mg with no DLTs. At the 37.5mg level, one patient had a DLT (wound dehiscence) and another three of five patients experienced Grade 3 neutropenia. With 4 of 6 patients experiencing grade 3/4 toxicity, enrollment was stopped, and no further dose escalation was attempted. No patient had a CR or PR, but five patients (26%) had stable disease for >4 cycles. Grade 3/4 toxicities during cycle 1 were: neutropenia (16%), leukopenia (16%), wound complications (5%), and abdominal pain (5%). PK analysis showed significant inter-patient variability of sunitinib and S-M. There were no PK alterations of sunitinib between the arms, but there were significant alterations in the PK of S-M. Efavirenz resulted in a 220% increase, whereas ritonavir caused a 69% decrease in the AUC of S-M, respectively.

## Conclusion

The recommended dose of suninitib for patients on ritonavir is 37.5mg, whereas patients on NNRTI-based therapy can be treated with the standard dose of 50mg per day.

